# Climate-Triggered Insect Defoliators and Forest Fires Using Multitemporal Landsat and TerraClimate Data in NE Iran: An Application of GEOBIA TreeNet and Panel Data Analysis

**DOI:** 10.3390/s19183965

**Published:** 2019-09-14

**Authors:** Omid Abdi

**Affiliations:** Institute for Cartography, Department of Geosciences, Faculty of Environmental Sciences, TU Dresden, 01069 Dresden, Germany; omid.abdi@mailbox.tu-dresden.de; Tel.: +49-351-463-33568

**Keywords:** GEOBIA, TreeNet, insect infestation, defoliators, Landsat 8 OLI, TerraClimate, climate hazards, drought, forest fires, panel data

## Abstract

Despite increasing the number of studies for mapping remote sensing insect-induced forest infestations, applying novel approaches for mapping and identifying its triggers are still developing. This study was accomplished to test the performance of Geographic Object-Based Image Analysis (GEOBIA) TreeNet for discerning insect-infested forests induced by defoliators from healthy forests using Landsat 8 OLI and ancillary data in the broadleaved mixed Hyrcanian forests. Moreover, it has studied mutual associations between the intensity of forest defoliation and the severity of forest fires under TerraClimate-derived climate hazards by analyzing panel data models within the TreeNet-derived insect-infested forest objects. The TreeNet optimal performance was obtained after building 333 trees with a sensitivity of 93.7% for detecting insect-infested objects with the contribution of the top 22 influential variables from 95 input object features. Accordingly, top image-derived features were the mean of the second principal component (PC2), the mean of the red channel derived from the gray-level co-occurrence matrix (GLCM), and the mean values of the normalized difference water index (NDWI) and the global environment monitoring index (GEMI). However, tree species type has been considered as the second rank for discriminating forest-infested objects from non-forest-infested objects. The panel data models using random effects indicated that the intensity of maximum temperatures of the current and previous years, the drought and soil-moisture deficiency of the current year, and the severity of forest fires of the previous year could significantly trigger the insect outbreaks. However, maximum temperatures were the only significant triggers of forest fires. This research proposes testing the combination of object features of Landsat 8 OLI with other data for monitoring near-real-time defoliation and pathogens in forests.

## 1. Introduction

Despite prosperous traditional approaches such as dendrological assessment and field observations for identifying driving forces of insect outbreaks from individual tree to stand scales [1], remotely sensed approaches are extensively progressing either for delineation insect-infested objects or for the mensuration of infestations induced by abiotic and biotic agents throughout forest biomes [2,3,4,5]. However, some novel algorithms for data mining and machine learning such as TreeNet [6] for delineation insect-infested objects from non-insect-infested objects of images, some high-resolution climate data such as TerraClimate [7] for assessing drought and climate hazards dimensions, and some associations such as interactions between insect outbreaks, forest fires, and climate hazards have received less attention in earlier studies. 

Although monitoring the bark beetle infestation and coniferous defoliation are dependent on high-resolution and multi-spectral images [8,9], detecting broadleaved defoliation has been predestined by the spectral–temporal information of images, even by single near-infrared-derived vegetation indices of images with high-temporal resolutions [10]. Moreover, Landsat images have indicated high accuracy for detecting forest-infested patches using either classification algorithms in a specific date [11] or by applying multitemporal spectral-derived indices [12,13,14]. Moderate stages of tree infestation are significantly discernible through multitemporal spectral indices, while severe infestation is highly discernible through classification approaches [15]. 

Several number of studies exerted data mining and machine learning algorithms such as random forest [4,16,17] and decision tree [18] or maximum likelihood classification [19,20] to discern insect-defoliated from non-insect-defoliated forests. However, numerous remote sensing-derived indices are growing for quantifying the insect-induced defoliation intensity in terms of the long-term archive of Landsat products [13,14,18]. For example, Townsend et al. [13] claimed that Landsat-derived near-infrared (NIR) and short-wave infrared (SWIR) indices such as the normalized different infrared index (NDII) and the moisture stress index (MSI) were superior to the Landsat-derived red and NIR indices such as the normalized difference vegetation index (NDVI) for estimating the defoliation induced by *Lymantria dispar* in the oak forests at five different times. Similarly, Rullán-Silva et al. [14] examined the efficiencies of 10 Landsat-derived vegetation indices for estimating the defoliation induced by *Rhynchaenus fagi* in the beech forests. They concluded that the MSI showed the highest correlation with the intensity of defoliation with respect to the mixed-effects model. However, the attitude of analyzing meaningful image-objects for the classification of infested and non-infested forests [21] by the cooperation of image-derived features and GIS-based methods and databases is developing, that is, Geographic Object-Based Image Analysis (GEOBIA) [22]. In addition, earlier results of studies have demonstrated that the combination of object features derived from medium-resolution images such as Landsat and SPOT with ancillary data such as topography has increased the precision of discerning forest infestations induced by bark beetle [8]. Machine learning algorithms such as random forest improved the classification efficiency with image-derived object features for mapping forest infestations [23]. However, evaluating the efficiency of combination GEOBIA and other machine learning methods such as TreeNet for mapping infested forests induced by defoliators has not been addressed up to now.

Forests are mainly degraded by interactions between abiotic agents such as high temperatures, drought, moisture variability, anthropogenic interventions, and forest fires and biotic agents such as invasive species, tree mortality, insect infestations, and pathogens [24,25,26,27]. The abiotic agents can trigger the effects of biotic agents, and their synchronization significantly results in tree species’ hydraulic deterioration and carbon starvation [11,24,25,27]. Climate change studies demonstrated that defoliators’ population rate during a growing season is correlated to the temperature condition of its hydrological year [28]. Temperature increases can enable insects to survive during the cold season [29,30,31] and provide rich sources of nutrition from the mature leaves by changing the trees phenology cycle; along with that, they can also increase the risk of insect outbreaks during the growing season [32]. However, there is existing uncertainty about the effects of droughts on the insect outbreaks in the forest biomes [33]. Increasing the droughts’ dimensions may result in providing conditions for insect outbreaks [11], tree mortality [34], or increasing the forest fires severity [35]. However, serious effects of insect defoliators emerge during the moderate drought condition or wetter seasons following droughts occurring [11,24,33,36]. Changes in moisture capacity regarding either high moisture availability [1,37] or low moisture availability [38] are identified as the main driver forcing of some insect outbreaks. The synchronization of droughts following the conditions of above-average moisture availability may result in providing an appropriate condition for insect outbreaks [24] as well.

Mutual interactions between insect infestations and forest fires were documented in some studies [39]. Insect infestations may affect the fuel loads of the tree species and increase the severity of forest fires at landscape-level scales [34,39,40]. However, some studies reported declining the severity of forest fires by increasing the mortality induced by insect attacks [41] or neutral effects of insects and pathogens on the fire characteristics [42,43] particularly in the coniferous forests. Additionally, there is evidence of the probability of increasing the risk of insect outbreaks in those trees that were damaged or weakened following a low severity of forest fires [44] or in fire-induced larger patches of canopy cover [45]. 

The Caspian Hyrcanian broadleaves and mixed forests have been degrading, as a virgin ecoregion of the temperate forests’ biome, by a variety of biotic and abiotic agents such as deforestation [46], forest fires [47], drought [48], and climate hazards with consequences of massive tree mortality [49] in northeast (NE) Iran during recent decades. Droughts’ dimensions could significantly affect the water content and greenness properties of Hyrcanian forests based on the MODIS-derived normalized difference water index (NDWI) and NDVI [48,49]. The stages of moderate to extreme tree mortality events showed a significant association with the high intensity of forest water content deficit derived from the MODIS–NDWI; however, the severe defoliation only showed a significant relationship with the intensity of forest greenness loss derived from NDVI in NE Iran [49]. In addition to climate hazards, drought, and forest fires, there has been rising concerning reports about the outbreaks of some insect defoliators such as *Lymantria dispar*, *Erannis defoliaria*, and *Operophtera brumata* [50,51] as well as pathogens [52,53,54] throughout Hyrcanian forests during recent years. Therefore, this study used TreeNet to delineate insect-infested forests from non-insect-infested forests using numerous Landsat 8 OLI-derived object features, topographic-derived features, and tree species types in Hyrcanian forest, NE Iran. Moreover, it will explore the mutual relationships between the intensity of insect infestation and the severity of forest fires in the presence of TerraClimate-derived climate hazard variables for the period of time of insect outbreaks and forest fires within the TreeNet-derived insect-infested forest objects.

## 2. Materials and Methods 

### 2.1. Study Area

**Figure 1:**
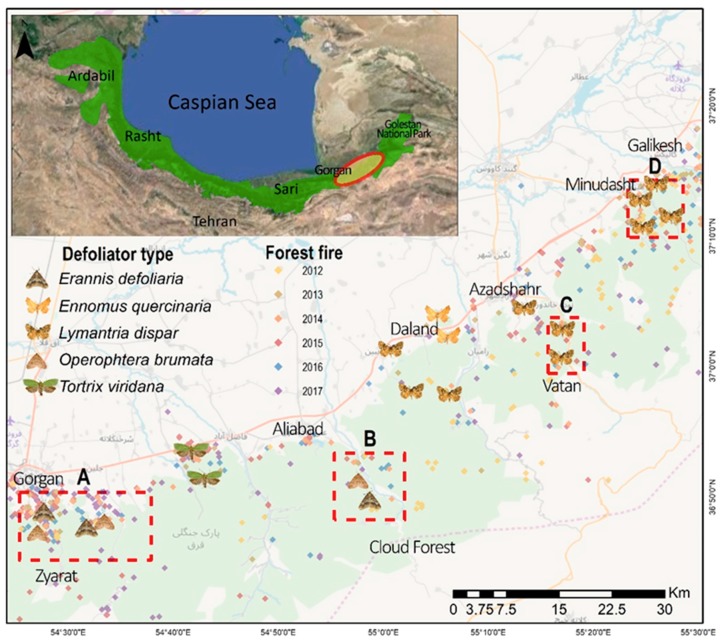
Study area in the Hyrcanian forest ecoregion in the southeast Caspian Sea. Spatial scatter of the insect defoliators and forest fires samples of 2012–2017 in northeast (NE) Iran. The eastern part of the study area was affected by *Lymantria dispar*, while the western area was affected by *Erannis defoliaria* and *Operophtera brumata.*

The eastern forests of the Hyrcanian ecoregion were selected for this research. This area is extended from Gorgan to Galikesh in the Golestan province, NE Iran (Figure 1). These forests comprise a mixture of broadleaved tree species such as *Quercus castaneifolia*, *Fagus orientalis*, *Carpinus betulus*, *Acer spp.,Tilia platyphyllos*, and *Parrotia persica* [55]. The western parts were infested by the defoliators of *Erannis defoliaria* and *Operophtera brumata*, while the eastern parts were affected by *Lymantria dispar* (Figure 1). Moreover, the frequency of forest fire events has been increasing in this region during recent years (Figure 1).

### 2.2. Data and Field Mensuration

**Figure 2:**
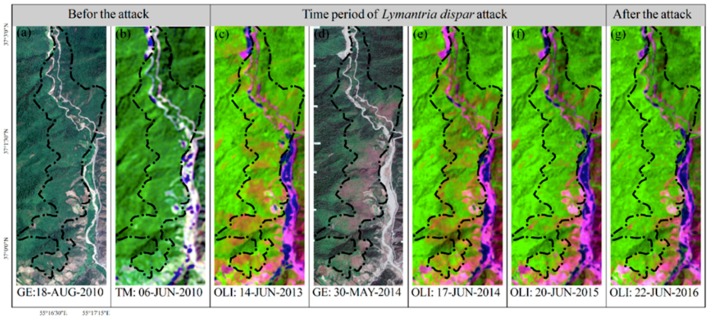
Site C (Figure 1): The images of Google Earth (GE) [59] and Landsat 5 TM (SWR, NIR, R) show that the forest was in a healthy condition before the attack of *Lymantria dispar* in 2010 (**a**, **b**); the symptoms of the infestation emerged on the Landsat 8 OLI (SWR1, NIR, R) as “olivenite green” in 2013 (**c**); the insect significantly infested the region based on the GE (**d**) and Landsat 8 OLI (**e**) images in 2014, while it declined in 2015 (**f**) and ended in 2016 (**g**).

This study identified the insect-infested regions from available reports, local media, and field observations. The attributes of defoliated spots including the type of defoliators, the position, the dominant type of host tree-species, and the evidence of current and previous egg laying were documented. The accurate spatial extent areas of the defoliation were delineated using time-series composite bands of Landsat 8 OLI [56] and the images of Google Earth (Figure 2) coincided with the advanced larval stage of insect defoliators (Figure 1). A peak of attack was recognized in 2014; the infested objects for this time were delineated from the healthy forest using GEOBIA and TreeNet. The forest fires data were achieved from field surveying, local media, and the available historical database that were provided by the Department of Natural Resources and Watershed Management of Golestan province from 2010 to 2017. The burnt area, duration, and frequency of forest fires were used during a fire season to reach the forest fire severity of a specific location.

We used monthly TerraClimate data for calculating [7] long-term dimensions of anomalies of drought, temperature, and soil moisture from 1987 to 2017. We derived the annual intensity hazard of climate variables from their dimensions as causes of triggering insect attack and forest fires for modeling by the panel data approaches (see Section 2.3.3). The ancillary data such as the Topographic Position Index (TPI), Terrain Ruggedness Index (TRI), and Topographic Wetness Index (TWI) were derived from the ALOS PALSAR data elevation [57] for the study area. Forest types were vectorized concerning the scanned maps of the forest management plans [58]. These data were used for mapping insect-infested forests along with Landsat 8 OLI data.

### 2.3. Methodology

#### 2.3.1. TreeNet-Based Insect Infestation Mapping

GEOBIA was used to delineate the insect-defoliated areas from the healthy forests through image segmentation and TreeNet classification. We derived image objects from a set combination of the main spectral and panchromatic channels of Landsat 8 OLI for the peak times of defoliation (May–June) through the multiresolution segmentation algorithm. To minimize the mean heterogeneity of image objects, we assigned optimal scale parameters by trial and error, with higher weights for the red, NIR, and SWIR bands; the compactness value of 1; and the shape value of 0 in the eCognition Developer 9 [60].

• Object features

Various object features (95 features) were derived from the main channels of Landsat 8 OLI, topography data, and forest types, as shown in Table 1. The object features were classified into four main groups including spectral features, surface texture features, geometric features, and the features derived from ancillary data in the GIS. A single database was created including all the derived features of segment objects, and was utilized for classification using TreeNet.

• TreeNet classification

The stratified random sampling method was used for selecting samples of insect-infested objects (defoliation >50%) and non-insect-infested objects for assessing the object features that control insect outbreaks and discriminate them from the non-insect-affected forests. The TreeNet algorithm was applied for determining influential variables depending on the test sets and generalizing the obtained scores to all feature objects to distinguish between the insect-infested and non-insect-infested objects. Classification in TreeNet is a particular form of regression that produces a possibility of response for a variable and accurately ranks the predictor variables based on their importance from “most likely” to “least likely” to the target variable. TreeNet creates boosting regression models through sequentially fitting a very small tree in several stages. Accordingly, each stage learns from the available training data by a specific learning rate [6]. 

We applied TreeNet in Salford Predictive Modeler 8.3 [75] to build our model with respect to its classification precision, working with both parametric and non-parametric variables, handling big data and missing datasets, connecting to GIS, plotting the univariate and bivariate relationships between the response and predictor variables through partial dependency plots (PDPs), yielding reliable results despite existing non-stationary in data, and ranking the predictor variables in terms of their importance in the model [6,76,77]. One-third of the samples were randomly assigned for the testing set, and the remaining were considered as the learning samples. We set the number of trees and maximum nodes per tree to 500 and 6, respectively. The shrinkage method was chosen as “auto” to eliminate the complexity of overfitting and to set the learning rate of the model, which was calculated to be 0.01. Moreover, we evaluated the number of optimal trees using the criterion of the area under the receiver operating characteristic (ROC) curve and the misclassification rate for the test samples. The confusion matrix was used to assess the performance of the classification model with respect to the test samples using four measures: (1) Sensitivity: the proportion of the insect-infested objects that are correctly classified, (2) Specificity: the proportion of the non-insect-infested objects that are correctly classified, (3) Precision: the proportion of the actual classified insect-infested objects divided by total number of the insect-infested class testing objects, and (4) an F1 statistic derived from the sensitivity and precision metrics as shown in Equation (1), in which its values close to one indicate a high sensitivity of the model:

(1) $$F1\;statistic=\frac{2\left(Sensitivity\times Precision\right)}{Sensitivity+Precision}$$

#### 2.3.2. Intensity of Insect Infestation, Severity of Forest Fire, and Climate Hazards

The intensity of defoliation was retrieved by analyzing the long-term deficit of NDWI-derived forest-water content within the TreeNet-derived insect-infested polygons. We calculated the dimensions of defoliation including the severity, frequency, and duration of the yearly defoliations from the anomalies of the Landsat NDWI from 1987 to 2017. The yearly hazard intensity of defoliation was mapped concerning the introduced approach by Abdi et al. [49], which is a combination of standardized values of defoliation dimensions through the fuzzy gamma operator [78].

We obtained the spatial variations and characteristics of daily forest fires from diverse resources from 2010 to 2017. Several studies verified the strength of Landsat-derived burn severity indices such as the differenced Normalized Burn Ratio (dNBR) [79] and the Relativized dNBR (RdNBR) [80] for large fires; however, some fires’ characteristics such as size area, duration, and the type of fire may restrict these indices applications for post-fire monitoring in northeast Iran. Therefore, the severity of forest fires was calculated from the combination of the ground-based characteristics of fires including the frequency, size, and duration of fires within segment objects. The fire characteristics’ values were standardized between zero (low) and one (high) by exerting the membership function of fuzzy linear [81]. The severity of forest fires was obtained by overlaying the standardized layers of fire characteristics using the fuzzy gamma operator [78] during a fire season.

TerraClimate data were applied to model the hazard intensity of climate variables throughout the hydrological years (October to September) from 1987 to 2017. The features (i.e., severity, frequency, and duration) of yearly anomalies of the climate variables were combined to obtain the hazard intensity indices of drought, maximum temperatures, and soil moisture deficit using the fuzzy gamma operator [78] as well.

#### 2.3.3. Relationships among Insect Infestation, Forest Fires, and Climate Hazards

We examined mutual relationships between insect infestation and forest fires in the presence of the hazard intensity of climate variables within the insect-affected objects using the panel data models. To include both the spatial and time effects of the data, we performed the panel data models [82] for the estimation of the intensity of defoliation affected by forest fire severity and climate hazards (Equation (2)), as well as the severity of forest fires induced by insect defoliation and the climate hazards (Equation (3)):

(2) $$Insect\;infestation=fn\left(F_{t-1},\;D_{t},\;D_{t-1},\;T_{t},\;T_{t-1},\;S_{t},\;S_{t-1}\right)$$

(3) $$Fire\;severity=fn\left(I_{t},\;I_{t-1},\;D_{t},\;D_{t-1},\;T_{t},\;T_{t-1},\;S_{t},\;S_{t-1}\right)$$

where *I_t_*, *F_t,_ D_t_*, *T_t_*, and *S_t_* are the averages of insect infestation intensity, fire severity, drought intensity, high temperature, and soil moisture deficits in the current year (*t*); and *I*_*t* − 1_, *F*_*t* − 1_, *D*_*t* − 1_, *T*_*t* − 1_, and *S*_*t* − 1_ are the averages of these variables for the previous year (*t* − 1), respectively.

##### Panel data models

Analyzing the panel data was established based on the combination of multiple observations for the same cross-sections and time series, which is written in a standard model as shown in Equation (4) [83]:

(4) $$y_{i,t}=\beta^{\prime }X_{it}+Z_{i}\alpha +\varepsilon_{it}$$

For i=1, 2,3,…,N and t=1, 2,3,…,T; where *N* stands for the number of individuals (cross-sections), and *T* is the number of times. The vector $X_{it}$ refers to the *k* regressors (Equations (2) and (3)). The vector $\beta^{\prime }$ refers to unobserved coefficients, which need to be estimated. The term $Z_{i}\alpha$ expresses the cross-section specific effects. The error terms of the model were indicated by $\varepsilon_{it}$. Three panel data models are defined regarding the nature of included variables in the vector $Z_{i}$, including the common effects, fixed effects, and also random effects models (Equations (5)–(7)). 

The common effects model does not consider time and cross-sectional effects. The vector $Z_{i}$ contains only one constant coefficient: α. The coefficients of this model are estimated by applying the ordinary least squares (OLS) approach:

(5) $$y_{i,t}=\beta^{\prime }X_{it}+\alpha +\varepsilon_{it}$$

The fixed effects model assumes specific cross-sectional effects from different intercepts to obtain unobserved heterogeneity. The parameter of fixed effect $\alpha_{i}$ is constantly estimated over time with the estimators’ normality assumption. This model assumes that cross-sectional effects are correlated with the included regressors $X_{it}$:

(6) $$y_{i,t}=\beta^{\prime }X_{it}+\alpha_{i}+\varepsilon_{it}$$

The random effects model assumes that unobserved cross-sectional heterogeneity is not correlated to the included regressors $X_{it}$. The coefficients of this model are estimated using the generalized least squares (GLS) estimator:

(7a) $$y_{i,t}=\beta^{\prime }X_{it}+\alpha +\mu_{it}$$

(7b) $$\mu_{it}=\alpha_{i}+\varepsilon_{it}$$

The random effects model is preferable to the fixed effect model if samples were taken from a larger population and the estimations’ generalization to the population was aimed. The higher accuracy of estimates and greater flexibility of this model in comparison with the fixed effects model were reported [84,85] as well.

##### Testing for fixed effects and random effects

Tests were accomplished to make a distinction between panel data models. The Chow test was used for testing fixed effects against common effects [83], which determines whether a single regression model (common effects) is superior to the two separate regression models (fixed effects). The Hausman’s specification test [86] was used for selecting whether the fixed effects model or the random effects model is appropriate [87]. The Hausman test makes a distinction between a model that assumes its cross-sectional effects are not correlated with its regressors (random effects), and a model that assumes that these relationships are established (fixed effects) [83]. 

## 3. Results

### 3.1. Insect Defoliation Mapping

The highest performance of TreeNet was obtained after the formation of 333 trees with the optimal ROC value of 93.4% for discriminating insect-infested and non-insect-infested forest objects. The model results using 83 out of 95 predictor variables yielded an average correctness of 87% for testing samples to predict the insect-infested and non-insect-infested forests (Table 2). 

**Figure 3:**
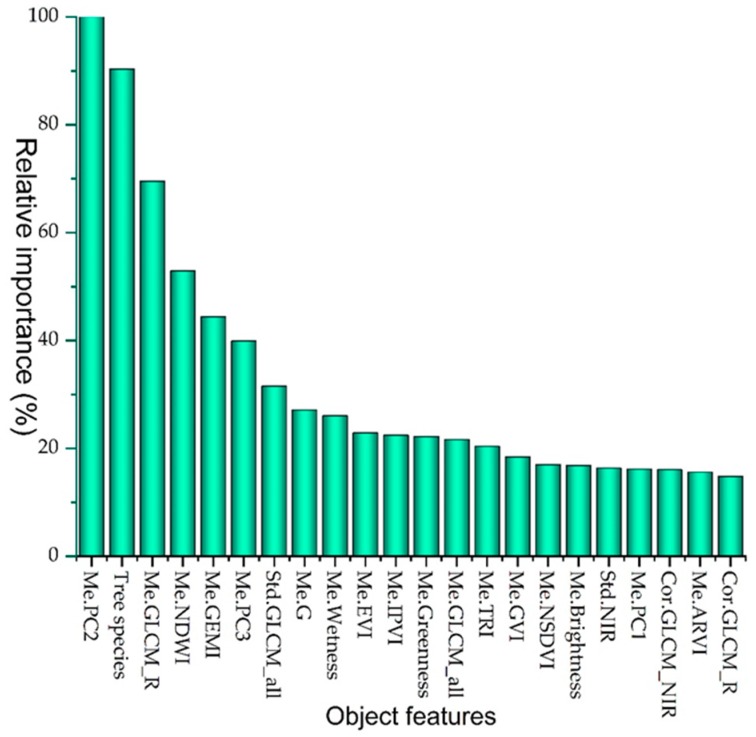
The importance of top predictor variables of Landsat 8 OLI and ancillary features for discerning insect-infested from the non-insect-infested objects using TreeNet. The most important predictor variable (i.e., the mean of the second principal component, or PC2) has gained a value of 100%, and other features were rescaled based on their importance relative to the PC2.

**Figure 4:**
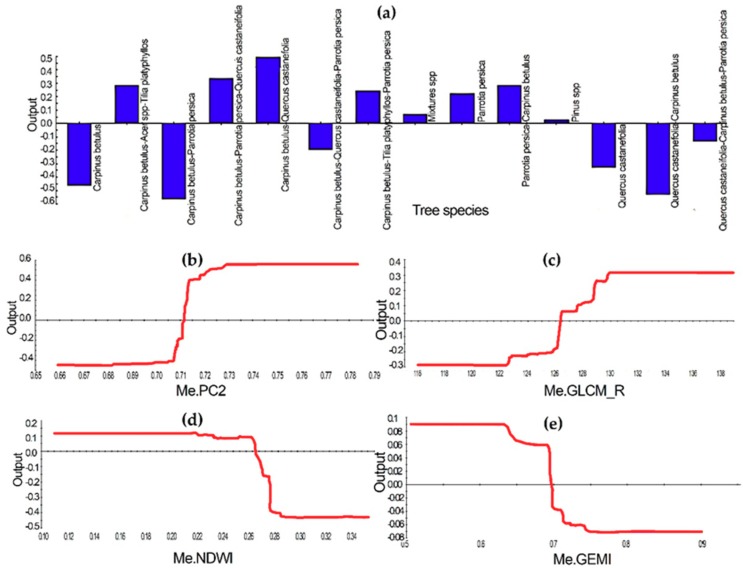
Univariate partial dependency graphs for the top five-predictor variables for classification of insect-infested forests in NE Iran. Positive values of the outputs indicate a direct association of a class of the categorical variables or values of the continuous variables with the focus class. Eight tree species showing positive relationship with the infested class (**a**), the mean of PC2 and the mean of red band derived from GLCM show a positive association at values of greater than 0.711 (**b**) and 126.30 (**c**), and the mean values of the NDWI and GEMI show a positive relationship with the presence of infestation until the values of 0.267 (**d**) and 0.697 (**e**).

Relative variable importance values describe that the top predictors are the mean of PC2, tree species, and the mean of the red channel derived from the gray-level co-occurrence matrix (GLCM), NDWI, and global environment monitoring index (GEMI) (Figure 3). The positive log-odds values of the insect-infested class were significantly increased when the mean of the PC2 values had passed the point of 0.70. Tree species with the domination of *Carpinus betulus-Quercus castaneifolia* and *Carpinus betulus-Acer spp.-Tilia platyphyllos* exhibited the highest partial relationships with the insects’ presence. The average log-odds values of the insect-infested class were increased from –0.287 to 0.314, where the mean of the red band derived from GLCM ranged from 122 to 130. The average log-odds dropped at values of 0.264 and 0.635 of the NDWI and GEMI, respectively, where the probability of infestation also reduced (Figure 4).

**Figure 5:**
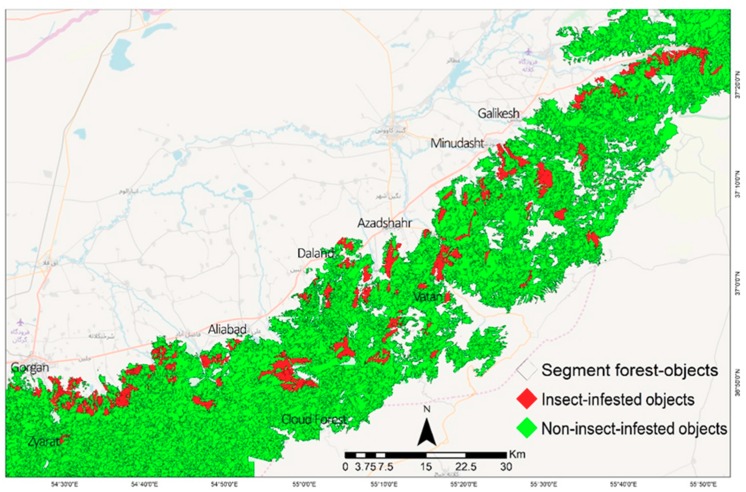
The insect-affected and non-insect-affected forest-objects derived from the influential object features of Landsat 8 OLI and ancillary data using Geographic Object-Based Image Analysis (GEOBIA) and TreeNet in the Hyrcanian region, NE Iran.

The TreeNet model was rebuilt with respect to the top influential object features that have gained a minimum score of 15% importance relative to the most important variable. The insect-infested and non-insect-infested map was created with the contribution of the top 22 predictor variables over the study area (Figure 5).

**Figure 6:**
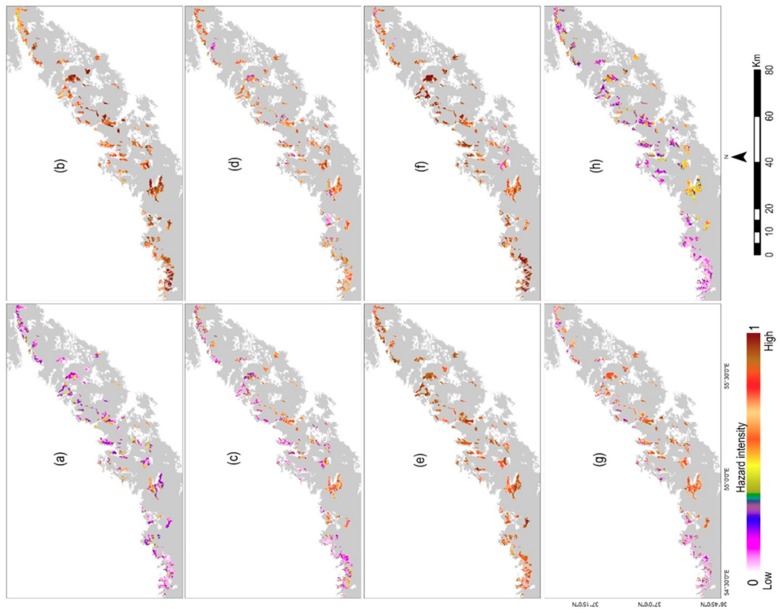
The yearly hazard intensity of defoliation for the time period of 2010 to 2017 (**a–h**) within the insect-infested forest objects. The monthly anomalies of NDWI were obtained from the time series of Landsat 5, 7, and 8 from 1987 to 2017. The dimensions of forest water content deficit including severity, frequency, and duration were derived from Landsat–NDWI anomalies for the time period of defoliation (2010–2017). The values of these dimensions were standardized by the membership functions of fuzzy linear and fuzzy large between zero and one. The hazard intensity of defoliation was obtained by overlaying the standardized layers of dimensions of the NDWI anomalies using the fuzzy gamma operator within the insect-infested segment objects during a growing season in Hyrcanian forests, NE Iran.

The hazard intensity of defoliation derived from the time-series anomaly of Landsat–NDWI was mapped as an insect infestation indication within the delineated insect-infested forest objects from 2010 to 2017 (Figure 6). The maps demonstrated that the infestation intensity was considerable in 2011, 2014, and 2015 (Figure 6b,e,f).

### 3.2. Insect Infestation, Forest Fires, and Climate Hazards Modelling

The results of the Hausman test indicate that the random effects model was superior to the fixed effects model for the variables expressing that control either the intensity of insect infestation (*X*^2^ = 11.87, df = 7, p= 0.105) or severity of forest fires (*X*^2^ = 6.72, df = 8, p = 0.567). Furthermore, the results of the Chow test showed that the fixed effects model was superior to the common effects in both models (*X*^2^ = 37.39, df = 18, p = 0.004; *X*^2^ = 43.31, df = 18, p = 0.000). 

The summary of the random effects model reveals that the drought condition (SPI), maximum temperature (Tmax), and the deficit of soil moisture of the current year—along with the maximum temperature, high soil moisture availability, and the severity of forest fires of the previous year—were the significant variables, which are controlling the insect infestation intensity during 2010–2017 in the study area (Table 3). However, the intensity of drought of the previous year was not significant, but its coefficient is positive. Although the majority of climate hazards demonstrated positive coefficient with the severity of forest fires, Tmax of the current year (β = 0.330, p < 0.05) and Tmax of the previous year (β = 0.196, p < 0.01) were the only significant variables. The insect infestation (II) of the previous year showed a positive relationship with the severity of forest fire as well; however, it is not statistically significant (β = 0.106, p > 0.05) in NE Iran (Table 4).

## 4. Discussion

This study exerted novel remote sensing-based data collections (e.g., Landsat and TerraClimate) and approaches (e.g., TreeNet and panel data models) in order to discern insect-infested forests and quantify the multitemporal intensity of forest defoliation, climate hazards, drought, and the severity of forest fires along with modeling their temporal and spatial interactions in NE Iran.

We obtained reliable discrimination between insect-infested forests and non-insect-infested forests (Table 2) using the important collections of Landsat 8 OLI-derived and ancillary object features using the TreeNet algorithm. The summary of variable importance represented that the object features extracted from the Landsat channels show higher performance in comparison with the main spectral channels of Landsat (Figure 3). For example, the highest value recorded for the mean of PC2, and after that the mean of red channel derived from GLCM and the mean values of vegetation indices, including the NDWI and GEMI, respectively. Analyzing one partial dependency revealed that the probability of the presence of defoliation increases along with the increase of average values of the PC2 and the mean of the red channel derived from GLCM, while it decreases by the increase of values of the NDWI and GEMI (Figure 3). The PC2 was positively loaded on the visible bands of Landsat 8 OLI with a higher coefficient for the red and green bands, which high-defoliated forest objects have obtained high values on PC2 (Figure 4b). The increase in the reflectance of some ranges of visible wavelengths due to the vegetation stress was reported in earlier studies [88,89], which are near to the green and red bands of Landsat 8 OLI. In contrast, PC2 was loaded negatively on the NIR and SWIR bands, as the objects with high biomass showed lower values on the PC2. While our analysis indicated that the PC2–Landsat 8 OLI is the top variable predictor, the third principal component (PC3) was positively loaded on the red band, and has showed considerable importance value in discriminating between insect-infested forest objects and healthy forest objects (Figure 3) as well. In addition, the ability of PC3 was reported for identifying year-to-year forest defoliation by *Lymantria dispar L.* using the time series of SPOT [90]. Moreover, the importance of Landsat-derived indices was presented for detecting insect-affected forests for different types of insects in other biomes [13,14,18]. This study results confirm that vegetation indices with respect to the NIR–SWIR (i.e., NDWI) are superior to those vegetation indices that are depending on the visible NIR channels for the delineation of insect-infested forest objects [13,14] in the Hyrcanian ecoregion as well.

Insect defoliators can affect the structure of vegetation [91]; therefore, image-derived textural attributes are appropriate for detecting insect-defoliating forest objects. However, the mean of the red channel derived from GLCM was among the top five predictors; Figure 3 indicates that about one-third of effective predictor variables are categorized in the first-order or second-order textural attributes for detecting the defoliation induced by insects in NE Iran. The superiority of image textures derived from GLCM and gray-level occurrence matrices (GLOM) [91,92] for detecting vegetation degradation induced by insect defoliation has been particularly demonstrated for high-spatial resolution images.

The results of variable importance describe that the type of tree species has a significant effect on the insect outbreaks as well. However, the partial dependence plot shows that mixed forest stands such as *Carpinus betulus* and *Quercus castaneifolia* with the other tree species types were highly correlated with the positive values of probability infestation in the Hyrcanian forests (Figure 4a). However, some studies reported insect defoliation in the monospecific stands in the other forest biomes [9,14,93]. 

The results of interactions between the intensity of insect defoliation with the intensity of climate hazards demonstrated that Tmax, drought, and soil moisture significantly increased the intensity of insect infestation. Both current and previous temperatures presented significant coefficients (Table 3). The high temperatures of the previous year can enable insects to survive during the winter [29,30,31] and change the cycle of forest phonology with the insect outbreaks consequences during the growing season [32].

Although some studies reported uncertainty about the effect of drought on the insect eruption [33], this study’s analyses confirmed that drought condition is a key trigger of increasing insect outbreaks in Hyrcanian broadleaved forests (Table 3). This area has experienced severe droughts from 2010 to 2011 [48]; however, the wetter seasons during 2012 and 2013 provided the condition for eruption insects during moderate drought seasons. Also, these fluctuations in the wet and dry seasons have been reported as a trigger of insect outbreaks [24]. The panel data models using random effects verified that the soil moisture availability [1,37] in the previous year significantly increased the intensity of insect infestation (Table 3). However, the effect of the deficit of soil moisture in the current year was not significant; its positive coefficient indicates that it was more likely that it intensified the insect eruption [38]. Therefore, the conditions of the previous year regarding its high temperatures and availability of moisture have supplied the sources of nutrients for insects and caused massive outbreaks in the current year with existing high temperatures and drought conditions. Earlier studies emphasized that high temperature is the main cause of increasing the intensity of tree mortality in the Hyrcanian forests [49].

Moreover, the random effects model indicated that the severity of forest fires of the previous year was a significant driver in increasing the intensity of insect infestation (Table 3). The trends of forest fires were dramatically increased following 2010 in the study area (Figure 1). Since the majority of the type of fires are classified as surface fires and single-tree burning in the study area, the likelihood of damaging trees [44] and opening fire-induced spaces [45] have increased the risk of insect infestation in these spots. The presence insect defoliators was higher in the locations with a high density of fires (Figure 1). In contrast, the intensity of insect defoliation either in the current year or in previous year was not a significant trigger for increasing the severity of forest fires in the Hyrcanian ecoregion (Table 4). The neutral effects of insect infestation on fire severity were demonstrated in the studies accomplished by Meigs et al. [42] and Kane et al. [43] as well. However, some researchers concluded that insect-induced tree mortality has decreased fuels and associated fire proneness, resulting in declining fire severity [41]. We can justify these results for two reasons in Hyrcanian forests: (1) insect infestations have not yet led to such a massive tree mortality that could load extensive fuels for fires [34,39,40] in this area, and (2) human activities are identified as the main causes of fires in this area, and mostly occurred in the condition of high temperatures [47]. This study confirmed that the maximum temperature of the current year and previous year are the only significant variables controlling forest fires in the Hyrcanian ecoregion (Table 4).

Landsat 8 OLI-derived features have shown reasonable efficiency for mapping insect-defoliated Hyrcanian mixed broadleaved forests for the investigated periods. Alternatively, this study proposes testing the capabilities of new data such as Sentinel-2 with higher spectral, spatial, and swat width that can produce dense time series, which are more appropriate for monitoring near-real-time insect infestation. There have been increasing reports about the outbreaks of other biotic agents such as bark beetles and pathogens in the Hyrcanian forests [52,53,54]; therefore, further investigations are required for identifying the spatial extent of these agents and exploring their triggers. Furthermore, separating mortality induced by insect defoliators from bark beetles or pathogens should be scrutinized.

## 5. Conclusions

This study has designed an object-based TreeNet framework to discern insect-defoliated forests using Landsat 8 OLI-derived and ancillary object features. Furthermore, we performed panel data models for quantifying the effects of TerraClimate-derived climate hazards on insect outbreaks and forest fires along with mutual associations of insect infestation and forest fires in the Hyrcanian broadleaved mixed forests, NE Iran. According to the analyses, we drew the following conclusions:

1. GEOBIA TreeNet indicated excellent performance with the contribution of Landsat 8 OLI-derived and ancillary object features for discriminating insect-defoliated forests from healthy forests.
2. Although the object features of Landsat 8 OLI recorded a higher importance for discriminating insect-defoliated objects, tree species has obtained the second rank of importance following the mean of PC2. In addition, other top image object features were the mean of the red channels derived from GLCM, the mean of NDWI, and the mean of GEMI, respectively.
3. The random effects model demonstrated higher performance in comparison with the fixed effects and common effects models to model mutual interaction of the intensity of insect defoliation and the severity of forest fire and their associations with the TerraClimate-derived climate hazards.
4. Maximum temperatures significantly triggered both insect outbreaks and forest fires. Although the drought conditions of the current year and the availability of soil moisture of the previous year were significant regarding the intensity of insect infestation, they have indicated neutral effects on the severity of forest fires.
5. The severity of forest fires of the previous year has triggered the intensity of insect infestation; however, the insect infestation was not effective for the forest fires.
6. Future studies will be required to explore the application of novel satellite images such as Sentinel-2 or the combination of Landsat 8 and Sentinel-2 for monitoring near-real-time insect-induced defoliation, identifying infestations resulting from bark beetles and pathogens, and discriminating between them.

## Figures and Tables

**Table 1:** Object features derived from Landsat 8 OLI channels and ancillary data (topographic and forest data) for discriminating defoliated forests from healthy forests in NE Iran.

Object features	Input data	Features ^1^ (No.)
Spectral features (32)	Blue, Green, Red, NIR, SWIR1, SWIR2	Mean (6), StdDev (6) of the spectral bands Mean and StdDev of spectral indices (14) (IPVI [61] GEMI [62], ARVI [63], GVI [64], NDVI [65], EVI2 [66], NDWI [67] Principal components (6) [68] Greenness (2), Wetness (2) [69], Brightness (1), Max. diff. (1)
Surface texture-features (56)	Single bands and all bands in all directions	GLCM_all dir._ (Homogeneity (7), Contrast (7), Dissimilarity (7), Entropy (7), Mean (7), Angle second moment (7), StDev (7), Correlation (7)) [70,71]
Geometric features (3)	Objects	Area (1), Compactness (1), Asymmetry(1) [71]
Ancillary data (4)	ALOS PALSAR Forest data	Topographic Wetness Index [72], Topographic Position Index [73], Terrain Ruggedness Index [74], Forest types

^1^ StdDev: Standard deviation; IPVI: Infrared percentage vegetation index; GEMI: Global environment monitoring index; ARVI: Atmospherically resistant vegetation index; GVI: Green vegetation index; NDVI: Normalized difference vegetation index; EVI2: Enhanced vegetation index 2; NDWI: Normalized difference water index; GLCM: gray-level co-occurrence matrix.

**Table 2:** Classification correctness of test samples for the TreeNet analysis to discriminate the insect-infested from the non-insect-infested forests in NE Iran.

Measure	Average	Overall accuracy	Specificity	Sensitivity	Precision	F1 statistic
Percent	87.15	86.76	80.56	93.75	81.08	86.96

**Table 3:** Results of panel data models for relationships between the intensity of insect infestation with the intensity of climate hazards of the current year and previous year as well as forest fires of the previous year in NE Iran.

Model	Constant	SPI*_t_*	SPI_*t* − 1_	Tmax*_t_*	Tmax_*t* − 1_	SoilM*_t_*	SoilM_*t* − 1_	Fire_*t* − 1_	*R* ^2^
Common effects	0.204	0.184 *	0.070 ^ns^	0.463 *	0.165 *	0.144 *	−0.134 *	0.171 *	0.680
Fixed effects	0.290 **	−0.039 ^ns^	−0.080 ^ns^	0.762 **	0.367 *	0.152 *	−0.072 ^ns^	0.154 *	0.798
Random effects	0.210 **	0.153 *	0.048 ^ns^	0.718 **	0.321 *	0.146 *	−0.126 *	0.194 *	0.706

** p value < 0.01, * p value < 0.05, and ns: not significant. SPI: standardized precipitation index; Tmax: maximum temperature; SoilM: soil moisture deficit; Fire: forest fire; *t*: current time; and *t* − 1: previous time.

**Table 4:** Results of panel data models for relationships between the severity of forest fires with the intensity of climate hazards and insect infestation of the current year and the previous year in NE Iran.

Model	Constant	SPI*_t_*	SPI_*t* − 1_	Tmax*_t_*	Tmax_*t* − 1_	SoilM*_t_*	SoilM_*t* − 1_	II*_t_*	II_*t* − 1_	*R* ^2^
Common effects	−0.0169 ^ns^	−0.085 ^ns^	0.030	0.385 *	0.177 *	0.032 ^ns^	−0.163 ^ns^	−0.059 ^ns^	0.105 ^ns^	0.210
Fixed effects	−0.032 ^ns^	−0.113 ^ns^	0.094 ^ns^	0.254 ^ns^	0.213 **	0.049 ^ns^	−0.126 ^ns^	−0.031 ^ns^	0.106 ^ns^	0.550
Random effects	−0.017 ^ns^	−0.101 ^ns^	0.117 ^ns^	0.330 *	0.196 **	0.041 ^ns^	−0.148 ^ns^	−0.020 ^ns^	0.106 ^ns^	0.236

** p value < 0.01, * p value < 0.05, and ns: not significant. SPI: standardized precipitation index; Tmax: maximum temperature; SoilM: soil moisture deficit; II: insect infestation intensity; *t*: current time; and *t* − 1: previous time.
